# A Weighted Estimation Approach to Correct for Imperfect Mutation Detection Due to Intra-tumor Heterogeneity in Tumor Sequencing

**DOI:** 10.64898/2026.10.02.756288

**Published:** 2026-10-08

**Authors:** Audrey Mauguen, Irina Ostrovnaya

## Abstract

Somatic mutations in sequenced tumors are often detected with imperfect sensitivity. They can be missed because tumor heterogeneity results in some mutations being absent in a sample at hand or present at very low frequencies which, combined with technical limitations like insufficient sequencing depth, reduces their detectability. It becomes apparent with multi-regional sequencing that some mutations confined to specific areas can be overlooked in single-region analyses. Adjusting for such sensitivity becomes critical when mutation frequencies between groups of subjects are compared, such as responders and non-responders to a given therapy. Otherwise, the estimate of the mutation prevalence, or of the difference in prevalences between the groups, can be biased, leading to inflated type I error. Here, we propose a weighted approach to adjust for imperfect sensitivity of the mutation detection and compared it to the unweighted approach. We assume that a surrogate of mutation sensitivity is observed only among patients with called mutation, and we derive its unconditional mean. Such adjustment leads to less biased estimates of mutational frequencies and better control of the type 1 error.

## Full Text Availability

The license terms selected by the author(s) for this preprint version do not permit archiving in PMC. The full text is available from the preprint server.

